# Achilles Tendon Xanthoma and Cholestanol Revealing Cerebrotendinous Xanthomatosis: A New Case Report

**DOI:** 10.1155/2021/6656584

**Published:** 2021-05-20

**Authors:** Mohamed Ahmed Ghassem, Aziza Mounach, Julien H Djossou, Hamza Toufik, Najlae El Ouardi, Lahsen Achemlal, Ahmed Bezza

**Affiliations:** Rheumatology Department, Military Hospital Mohammed V, Mohammed V University, Rabat, Morocco

## Abstract

Cerebrotendinous xanthomatosis (CTX) is an autosomal recessive lipid storage disease rarely reported in Africa. Therefore, we report a Moroccan first case report of CTX. A 20-year-old woman was presented in our department for bilateral swelling of the posterior aspect of ankles and the anterior aspect knees with gait disturbances evolving since the age of 7. The patient was the first child of consanguineous marriage. She had bilateral cataracts and developmental delay. Laboratory findings revealed that the plasma cholestanol level was remarkably elevated, and plasma and urine bile alcohol levels were elevated. MRI of ankles showed a bilateral diffuse thickening of the Achilles tendon with hypointense in T1 and heterogeneous hypersignal in T2 with spots in hypersignal in T1 and T2. Brain MRI revealed bilateral and symmetrical T2 hypersignal of dentate nuclei, without white matter signal alterations or cerebral or cerebellar atrophy. A biopsy obtained of the Achilles swelling with a histological study showed an aspect of tendon xanthoma. Hence, the diagnosis of CTX was made. MRI, especially brain MRI, plays an important role in the diagnosis of CTX.

## 1. Introduction

Cerebrotendinous xanthomatosis (CTX) is a rare autosomal recessive lipid storage disease, caused by mutations in CYP27A1 gene [1]. Incidence of this disease is the highest in Asians, followed by North Americans, Europeans, and Africans [2]. Since the first description in 1937, several hundred cases have been reported in the literature in Asia, in the United States of America, and in Europa, but little cases in Africa [3, 4]. To our knowledge, only one case was reported in Tunisia [5]. Therefore, we report a Moroccan first case report of CTX.

## 2. Case Presentation

A 20-year-old woman was presented in our department for bilateral swelling of ankles and knees. The patient was the first child of consanguineous marriage. She was delivered following a normal and term pregnancy with a normal birth weight and height. No history of atheromatous disease or intractable infantile-onset diarrheal was noted. At the age of 6, she developed blurred distance vision revealing a bilateral cataract and developmental delay and learning difficulties. At the age of 7, she presented bilateral swelling of the posterior aspect of the ankles worsen over time with apparition of bilateral swelling of the anterior aspect of the knees and gait disturbances.

**Figure 1:**
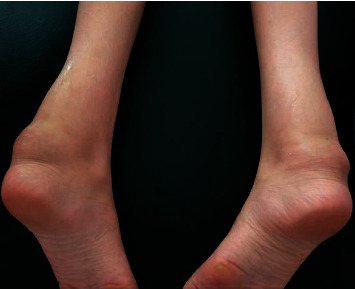
Bilateral and symmetrical hypertrophy of Achilles tendon.

Musculoskeletal examination revealed bilateral and symmetrical hypertrophy of Achilles tendons (Figure 1), quadriceps, and tibial tuberosities. Neurological examination revealed spastic gait and flascospastic paraparesis with proximal hypotonia and distal hypertonia. The osteotendinous reflexes were sharp and diffuse. A Babinski sign was present. The patient also presented with psychomotor slowdown. Superficial and deep sensitivities were normal. The rest of the physical examination did not reveal tuberous or skin xanthomas or xanthelasma.

Laboratory findings revealed that the plasma cholestanol level was remarkably elevated to 131 *μ*mol/L (reference range: 2–10), and plasma and urine bile alcohol levels were elevated. Reference range of plasma cholestanol matches those reported in the study of Gelzo et al. [6]. The analysis of plasma cholestanol was performed by gas chromatography coupled with a flame ionization detector (GC-FID). Total cholesterol, triglycerides, LDL-C, and HDL-C levels were normal. CYP27A1 gene mutation was not done due to lack of accessibility.

**Figure 2:**
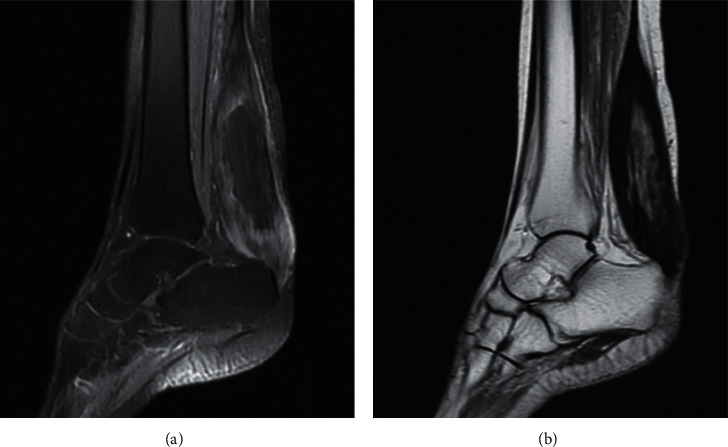
Sagittal images of the ankle MRI showing diffuse thickening of the Achilles tendon hypointense in T1 (a) and heterogeneous hypersignal in T2 (b).

**Figure 3:**
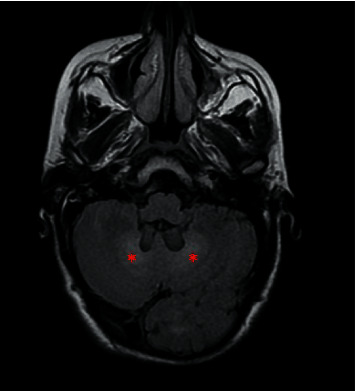
Axial image of brain MRI showing bilateral T2 hypersignal of the serrated nuclei (asterisks).

Ultrasound confirmed the origin of swelling by demonstrating a hypoechoic infiltration of the Achilles and quadriceps tendons, with loss of fibrillar structure. Magnetic resonance imaging (MRI) of ankles showed a bilateral diffuse thickening of the Achilles tendon with hypointense in T1 and heterogeneous hypersignal in T2 with spots hypersignal in T1 and T2 compatible with the appearance of tendon xanthoma (Figure 2). Brain MRI revealed bilateral and symmetrical T2 hypersignal of dentate nuclei not enhanced after injection of gadolinium, without white matter signal alterations or cerebellar atrophy (Figure 3).

A biopsy obtained of the Achilles swelling with a histological examination showed an aspect of tendon xanthoma made of fibroblastic tissue rich in cholesterol crystals; in contact with these crystals, numerous multinucleated giant cells were observed, associating numerous foamy histiocytes with them.

Finally, the diagnosis of cerebrotendinous xanthomatosis (CTX) was made by the combination of all these clinical findings, biochemical testing, histological examination, and radiological arguments. Chenodeoxycholic acid treatment (250 mg at a rate of 3 capsules per day) was offered to the patient.

## 3. Discussion

Cerebrotendinous xanthomatosis (CTX) is an autosomal recessive lipid storage disease, caused by mutations in CYP27A1 gene that result in production of a defective sterol 27-hydroxylase. Sterol 27-hydroxylase (CYP27A1) plays a key role in the synthesis of bile acids. CTX is responsible of an elevated plasma level of cholestanol and an accumulation of lipids in several tissues, particularly in the brain, eyes, and tendons [1]. This is a disease considered rare and probably underdiagnosed, especially in Africa due to the lack of technical facilities. Since the first description in 1937 by Bogaert, several hundred cases have been reported in the literature in Asia, in the United States of America, and in Europa, but little cases in Africa [3, 7]. To our knowledge, only one case was reported in Tunisia [5]. A genetic study, using a large cohort of adults from global populations, estimated incidence of CTX was the highest in South Asians and East Asians, followed by North Americans, Europeans, and Africans [2]. The prevalence of CTX is particularly high among Jews of Moroccan origin and Druze in Israel, i.e., 6/70,000 [4]. A Japanese nationwide survey published in 2018 identified 40 patients with CTX [8]. In the USA, the prevalence of CTX is estimated to be 3–5/100,000. In Europe, the prevalence of CYP27A1 mutation alone is 1/800,000 individuals in Spain and is approximately 1/50,000 in Caucasians [7, 9, 10]. Therefore, we report a Moroccan first case report of CTX.

The main limitation of our diagnostic approach was the absence of genetic analysis due to lack of accessibility. The gold standard for diagnosis of CTX is genetic analysis, but it is not obligatory in front of a clinicoimagery table suggestive associated an increase in cholestanol. A literature review carried by Wong et al. collected 194 CTX cases, with only 46% of patients performed a genetic analysis [11]. A genetic analysis was performed only in 35% of the 43 CTX patients, yielded by Duell et al. [12].

In front of a tendon xanthoma, we discussed the following hypotheses: familial hypercholesterolemia, sitosterolemia, and CTX. Familial hypercholesterolemia is an autosomal-dominant lipid storage disease caused by a deficiency in low-density lipoprotein (LDL) receptor activity. These abnormalities are responsible for the manifestly elevated LDL-cholesterol and triglyceride concentrations with the normal plasma cholestanol level [13]. Sitosterolemia is a very rare inherited sterol storage disease; it is characterized by extensive tuberous and tendon xanthomas, premature atherosclerosis, haemolytic anemia, arthritis, thrombocytopenic purpura, high plasma phytosterol concentration, and normal to mildly elevated plasma cholesterol levels [14].

The clinical presentation of CTX is heterogeneous encompassing various neurological and nonneurological manifestations, appearing in infancy or adulthood. It includes premature bilateral cataracts (88% of cases), intractable chronic diarrhea (50% of cases), tendon xanthomas (69% of cases), and progressive neurological signs (pyramidal tract signs in 77% of patients and cerebellar signs in 62% of patients) [1]. Tendon xanthomas most often affect the Achilles tendon, although it may also develop on the fingers and tibial tuberosities [1, 3].

The complete absence of sterol 27-hydroxylase (CYP27A1) leads to an increased excretion of bile alcohols in the faeces and urine and increased production of plasma cholestanol. Indeed, the elevation of plasma cholestanol and urinary bile alcohol levels is a characteristic of CTX [1, 3, 12].

Imaging plays an important role in the diagnosis. On the one hand, brain MRI is performant by showing a typically bilateral and symmetrical aspect orienting towards a metabolic origin. It is a bilateral T2 hypersignal of the dentate nuclei and white matter signal alterations with or without cerebral and cerebellar atrophy. On the other hand, MRI of ankles makes it possible to objectify the tendon xanthomas in the form of a fusiform thickening of the tendon with a heterogeneous signal [1, 3].

Early diagnosis of CTX is crucial because starting treatment with chenodeoxycholic acid early can improve neurological symptoms and even reverse the course of the disease. Diagnosis is based on clinical findings, biochemical testing, neuroimaging, and genetic analysis, which reveal the mutation of the CYP27A1 gene [1, 3].

## 4. Conclusions

In summary, the clinical presentation of CTX is heterogeneous and probably leading to an underdiagnosis of the disease, but knowledge of this presentation makes it possible to build/cultivate the habit of requesting a lipid assessment in young patients which will probably lead to an increase in diagnosed cases of CTX especially in the Maghrib region. Brain MRI which is a routine examination in front of any neuropsychiatric manifestations not explained by the brain scan plays an important role in the diagnosis of CTX.

## Data Availability

The data used to support the findings of this case report are included within the article.
